# SP-A binding to the SARS-CoV-2 spike protein using hybrid quantum and classical in silico modeling and molecular pruning by Quantum Approximate Optimization Algorithm (QAOA) Based MaxCut with ZDOCK

**DOI:** 10.3389/fimmu.2022.945317

**Published:** 2022-09-13

**Authors:** Sona Aramyan, Kirk McGregor, Samarth Sandeep, Angela Haczku

**Affiliations:** ^1^ If and Only If (Iff) Technologies, Pleasanton, CA, United States; ^2^ University of California (UC) Davis Lung Center Pulmonary, Critical Care and Sleep Division, Department of Medicine, School of Medicine, University of California, Davis, CA, United States

**Keywords:** SARS-CoV-2, SP-A, in silico, quantum computation (QC), glycosylation, immunoprotection, QAOA, MaxCut

## Abstract

The pulmonary surfactant protein A (SP-A) is a constitutively expressed immune-protective collagenous lectin (collectin) in the lung. It binds to the cell membrane of immune cells and opsonizes infectious agents such as bacteria, fungi, and viruses through glycoprotein binding. SARS-CoV-2 enters airway epithelial cells by ligating the Angiotensin Converting Enzyme 2 (ACE2) receptor on the cell surface using its Spike glycoprotein (S protein). We hypothesized that SP-A binds to the SARS-CoV-2 S protein and this binding interferes with ACE2 ligation. To study this hypothesis, we used a hybrid quantum and classical *in silico* modeling technique that utilized protein graph pruning. This graph pruning technique determines the best binding sites between amino acid chains by utilizing the Quantum Approximate Optimization Algorithm (QAOA)-based MaxCut (QAOA-MaxCut) program on a Near Intermediate Scale Quantum (NISQ) device. In this, the angles between every neighboring three atoms were Fourier-transformed into microwave frequencies and sent to a quantum chip that identified the chemically irrelevant atoms to eliminate based on their chemical topology. We confirmed that the remaining residues contained all the potential binding sites in the molecules by the Universal Protein Resource (UniProt) database. QAOA-MaxCut was compared with GROMACS with T-REMD using AMBER, OPLS, and CHARMM force fields to determine the differences in preparing a protein structure docking, as well as with Goemans-Williamson, the best classical algorithm for MaxCut. The relative binding affinity of potential interactions between the pruned protein chain residues of SP-A and SARS-CoV-2 S proteins was assessed by the ZDOCK program. Our data indicate that SP-A could ligate the S protein with a similar affinity to the ACE2-Spike binding. Interestingly, however, the results suggest that the most tightly-bound SP-A binding site is localized to the S2 chain, in the fusion region of the SARS-CoV-2 S protein, that is responsible for cell entry Based on these findings we speculate that SP-A may not directly compete with ACE2 for the binding site on the S protein, but interferes with viral entry to the cell by hindering necessary conformational changes or the fusion process.

## Introduction

**Figure 1:**
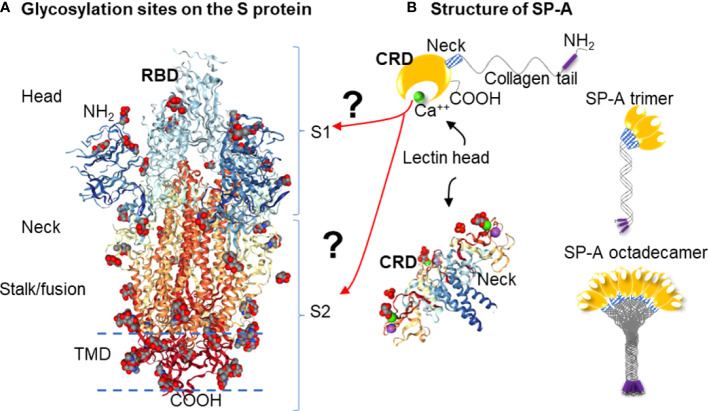
Hypothesis: SARS-CoV-2 Spike glycoprotein glycosylation sites are potential binding sites for SP-A **(A)**: Glycosylation sites on the SARS-CoV-2 spike glycoprotein trimer are denoted by NAG residues, shown in (O): red; (C): grey; (N): blue space fill balls. Structure of SARS-CoV-2 spike glycoprotein with a single receptor-binding domain up NGL Viewer (AS Rose et al. 2018) PDB: 6VSB DOI: 10.2210/pdb6VSB/ pdb EM Map EMD-21375: EMDB EMDataResource **(B)**: Structure of monomer (top panel), trimeric and octadecameric SP-A and potential carbohydrate recognition sites on the S protein by the Carbohydrate Recognition Domain (CRD) of the lectin head of SP-A. The CRD binds carbohydrate residues with high affinity in a Ca^++^ dependent manner. The *X-ray Crystal Structure* depicts the rat Surfactant Protein A neck and carbohydrate recognition domain ligated with mannose. Atoms represented by the spacefill balls are: (O): red; (C): grey; (Ca): green; (Na): purple. PDB: 3PAK DOI: 10.2210/pdb3PAK/ pdb 2010-11-03 Shang, F. et al. (X-RAY DIFFRACTION Resolution: 1.90 Å). (RBD, receptor binding domain; S1, Spike 1 region; S2, Spike 2 region; TMD, Transmembrane domain; CRD, Carbohydrate recognition domain).

The main site of the viral entry of the Severe Acute Respiratory Syndrome Coronavirus 2 (SARS-CoV-2) is through lung epithelial cells involving interactions between the Angiotensin Converting Enzyme 2 (ACE2) and the Spike glycoprotein (S protein) (1). The majority of enveloped viruses bind to host cell surface receptors *via* their surface glycoproteins. This process induces a conformational change of these viral ligands resulting in fusion with the host cell membrane delivering the virus genome to the cytoplasm (2). ACE2 as the main functional receptor was already identified for the SARS-CoV in 2003 when it was also established that the binding site (Receptor Binding Domain, RBD) was localized between amino acid residues 303 and 537 of the virus S protein (3, 4). The SARS-CoV and SARS-CoV-2 S proteins are highly similar and their structure together with their glycosylation sites have been partly established (4–8). The S protein is a trimeric class I fusion protein (**Figure 1**) with two functional subunits: S1 and S2. S1 is responsible for binding to the ACE2 receptor and S2 is responsible for host membrane fusion (4, 9–12). The S1 subunit RBD can be in a closed or an opened conformation. The open position is required for ACE2 binding. As demonstrated by numerous theoretical and experimental approaches (13–15), a main focus of research has been to find ways to interfere with S1 subunit RBD-ACE2 binding.

The S protein is highly glycosylated and in addition to ACE2 binding, it is known to ligate pattern recognition receptors. Each monomer in the S protein trimer has 22 glycosylation sites (shown in **Figure 1A**) (8, 16, 17). Glycosylation is important in protein conformation, target binding, and host evasion (7, 8, 18). The soluble carbohydrate pattern recognition receptors of the innate immune system could hinder ACE2 - S protein ligation through several different pathways including potential direct competition, inducing conformational changes that prevent receptor recognition, or sequestering the virus through opsonization for clearance by macrophages (19–21). The most abundantly expressed lung collectin, surfactant protein A (SP-A), is a particularly relevant pattern recognition host defense molecule because it is mainly produced by type II alveolar epithelial cells in the distal air spaces that are also the main site of entry for respiratory viruses (22–24). Together with SP-D and mannose-binding lectin (MBL), SP-A was implicated in binding to and regulating SARS-CoV-2 function (19, 21, 25–32).

Here we aimed to give *in silico* insights into the binding between SARS-CoV-2 S protein and SP-A. Since the RBD is much smaller in size than the entire S protein, this domain would provide an attractive reduced size target to be studied for binding predictions. However, whether SP-A directly binds to RBD, or other S1 or S2 areas, remains unclear. In fact, the RBD might be protected from SP-A access by glycosylation shielding (7). Further, targeted binding to the S2 fusion region was recently shown to effectively inhibit SARS-CoV-2 function (33–36), suggesting that the RBD may not be an exclusive target for viral inhibition. Importantly, SP-A preferentially targets carbohydrate moieties such as glycosylation sites that can be found in either the S1 or the S2 regions. For these reasons, we chose to assess the entire S protein in this study (**Figure 1A**).

Identification of the most likely binding sites between proteins found in Protein Data Bank (PDB) formatted files means that for “*n*” number of potential bindings between all potential atoms, “*n^3^*” or greater time in seconds is required for processing. While this could be straightforward when detailed binding kinetics data are available, it is difficult for proteins with no binding information, as chemical kinetics can require completion in non-deterministic, polynomial time. Such polynomial state computational problems can be infeasible or even impossible by classical computing (37, 38). Protein binding site analysis needs to be performed before docking assessment at the precision level of molecular dynamics, either by using third-degree polynomial topological algorithms (39) or quantum annealing devices that straddle the line between highly optimized classical computing and fully quantum computation within a first-degree polynomial complexity class (40, 41). Quantum Processing Units (QPUs) use the effects of quantum mechanics for methods of information transfer among bit-like devices (i.e., quantum bits, or “qubits”). Due to the qubits’ ability to hold multiple states, QPUs could ideally solve molecular kinetics calculations as they could represent every electron within a protein (42), given that QPUs can be treated as extra-large electrons due to their macroscopic quantum effects. Quantum processors provide superposition and entanglement features on their qubits and have the potential to take exponential scale problems and turn them into polynomial or even log scale problems. However, the largest QPUs that exist as per the writing of this paper are the Xanadu Borealis device with 216 qubits (43), the IBM Eagle with 127 qubits and the Google Bristlecone with 72 qubits (44). As one qubit simulates one extra-large electron (42), it would take many hundreds of qubits to model even the simplest proteins (45).

We, therefore, developed a model-simplification approach (QAOA-MaxCut) by systematically eliminating atoms within amino acids in the protein structure before processing for the selection of the most likely ones for binding. The QAOA-MaxCut protein pruning tool is based on the quantum approximate optimization algorithm (QAOA) (46) that includes further bioinformatics contextualization to aid the MaxCut algorithm. This hybrid approach combines the quantum computer’s ability to effectively solve exponential problems with a classical cost function to determine the best cuts within a set of quantum bits. If designed to scale effectively with classical devices, QAOA provides benefits with few qubits. In this study, we used a QAOA-MaxCut’s protein pruning tool running on a (QPU) connected to a 1-node classical computer for investigating the potential binding sites of SP-A to the SARS-CoV-2 S protein. This method was previously used in SARS-CoV-2 Spike-ACE2 complex pruning in Autodock Vina, investigating potential binding when azithromycin and hydroxychloroquine were considered for COVID-19 treatment, and was compared with GROMACS on the JUWELS and BRIDGES supercomputers for preparing a relaxed structure for docking (47, 48). Here we hypothesized that SP-A binds to the SARS-CoV-2 S protein and this binding interferes with ACE2 ligation by targeting the Receptor Binding Domain (RBD, **Figure 1A**).

## Materials and methods

### SP-A and S protein structures

We obtained the protein sequences and initial configuration data for the S protein and SP-A from the Research Collaboratory for Structural Bioinformatics (RCSB) protein data bank (**Figure 1**). The crystallographic coordinates for the SP-A protein structure were determined by available UniProt models. We selected 5FFR (49) as the most all-encompassing structural model of SP-A, covering 147 of its amino acids at a resolution of 2.20 Å. However, this model did include phosphocholine ligands, which could interfere with the direct probing of the amino acids that constitute the structure of SP-A (49). We therefore removed the phosphocholine ligands from the 5FFR model before binding site analysis. To investigate potential binding between the S protein and SP-A and to compare it with S protein-ACE2 binding in their respective sites and affinities, we completed a two-step analysis of the proteins’ crystallographic data, and further UniProt analysis of the results. The Protein Data Bank structure 6VSB was used to represent the SARS-CoV-2 Spike, as it had 44 of its 66 N-Acetylglucosamine (NAG) identified in experimental cryoEM microscopy work, not through computational placement (12). We would like to remark that since the initial release of the 6VSB model (that we used for identification of the NAG sites), the original NAG sites and identification numbers have been changed. Our data reflect the original NAG labeling numbers on this molecule.

### T-REMD with GROMACS

For the completion of the T-REMD analysis as a comparison to QAOA, GROMACS 5.0.4 was utilized to create a suitable solvent environment, along with a set of temperature and pressure controls, in order to most accurately determine the protein configuration in a binding environment. Being a molecular dynamics software, GROMACS completes sets of multi-axial nearest neighbor calculations for a set of forces for coordinate position and velocities across a number of time steps (50, 51). First, forces for each molecule within a solute and solvent are calculated using a prescribed set of forces unique to different solvation environments. To better understand a protein in a neutral solvent environment, we used three different force models: 1) the Assisted Model Building with Energy Refinement (AMBER) force field; 2) the Optimized Potentials for Liquid Simulations (OPLS) force field; and 3) the CHemistry At Harvard Macromolecular Mechanics (CHARMM) force field.

Being the oldest force field used, AMBER has the simplest form, with total potential energy for a macromolecule following a summation between bond energy as an ideal spring, geometrical energy from each angle within the covalent bonding between atoms, torsioning due to bond order, and intra-atomic forces represented as a van der Waals force added to an electrostatic force, wherein *f_ij_* represents the Fourier transformation, *E_ij_* represents the well depth of the atom’s location, and other constants represent their respective parts. This study used AMBER99sb.


(1) $$V(r^{N})=\sum_{i\in bonds}^{}k_{bi}{\left(l_{i}-l_{i}^{\text{o}}\right)}^{2}+\sum_{i\in angles}^{}k_{ai}{\left(\theta_{i}-\theta_{i}^{\text{o}}\right)}^{2}$$

(2) $$+\sum_{i\in torsions}^{}\sum_{n}^{}\frac{1}{2}V_{i}^{n}[1+\cos(nw_{i}-{ϒ}_{i})]$$

(3) $$+{\sum_{j=1}^{N-1}\sum_{i=j+1}^{N}f_{ij}\{\in_{ij}[{\frac{r_{ij}^{0}}{r_{ij}}}^{12}-2\frac{r_{ij}^{0}}{r_{ij}}}^{6}]+\frac{q_{i}q_{j}}{4\pi \in_{0}r_{ij}}\}$$

Equation 1. AMBER Force Field Formula (adapted from Case et al. AMBER9 Manual for Electrical Potential Across Protein.

OPLS (52) shares much of the same structure as AMBER. However, it aims to provide better analysis of the differences between bonded, nonbonded, and dihedral atoms, present on multiple energetic planes, through the use of torsional and electrostatic constants derived for each element and each organic functional group, represented as A and C. OPLS is also designed for use with the TIP3P water model, which is a 3-sided rigid water molecule with charges, as the default solvent for the force field.


(4) $$E(r^{N})=E_{bonds}+E_{angles}+E_{dihedrals}+E_{nonbonded}$$

(5) $$E_{bonds}=\sum_{bonds}^{}K_{r}{\left(r-r_{0}\right)}^{2}$$

(6) $$E_{angles}=\sum_{angles}^{}K_{\theta }{\left(\theta -\theta_{0}\right)}^{2}$$

(7) $$E_{dihedrals}=\sum_{dihedrals}^{}(\frac{V_{1}}{2}[1+\text{cos}(\phi -\phi_{1})]+$$

(8) $$\frac{V_{2}}{2}[1+\text{cos}(2\phi -\phi_{2})]+\frac{V_{3}}{2}[1+\text{cos}(3\phi -\phi_{3})]+\frac{V_{4}}{2}[1+\text{cos}(4\phi -\phi_{4})])$$

(9) $$E_{nonbonded}=\sum_{i>j}^{}f_{ij}(\frac{A_{ij}}{r_{ij}^{1}2}-\frac{C_{ij}}{r_{ij}^{6}}+\frac{q_{i}q_{j}}{4\pi \in_{0}r_{ij}}$$

(10 $$A_{ij}=\sqrt{A_{ij}A_{jj}}$$

(11) $$C_{ij}=\sqrt{C_{ii}C_{jj}}$$

Equation 2. OPLS Force Field Formula (adapted from Jorgensen et al.) for Electrical Field Across Protein (52).

CHARMM (53) is a force field (delete hyphen) model that aims to take OPLS further through the addition of an impropers and a Urey – Bradley term, which what both intend to improve upon the torsional modeling of the atomic interactions in OPLS through the accounting of bending and non-binding interactions between atoms in the 1,3 positions of an organic molecule due to proximity of electrostatic forces, respectively. This study used CHARMM36.


(12) $$V=\sum_{bonds}^{}K_{b}{\left(b-b_{0}\right)}^{2}+\sum_{angles}^{}K_{\theta }{\left(\theta -\theta_{0}\right)}^{2}+\sum_{dihedrals}^{}K_{\phi }(1+\text{cos}(n\phi -\delta ))$$

(13) $$+\sum_{impropers}^{}k_{\omega }{\left(\omega -\omega \right)}^{2}+\sum_{Urey-Bradley}^{}k_{u}{\left(u-u_{0}\right)}^{2}+$$

(14) $$\sum_{nonbonded}^{}(\in [(\frac{R_{\min_{ij}}}{r_{ij}}{)}^{12}-{\left(\frac{R_{\min_{ij}}}{r_{ij}}\right)}^{6}]+\frac{q_{i}q_{j}}{\in r_{ij}})$$

Equation 3. CHARMM force field formula adapted from Mackerell et al. for electrical potential across protein (53).

GROMACS software testing involved SP-A protein in water bulk that consisted of ~62,000 atoms (number of water molecules ~20,000) in a 6.5×6.5×6.5 nm^3^ cell. The test runs were done using the following parameters: 2fs timestep, PME electrostatics, and van der Waals forces truncated at 1.2 nm with corresponding pressure and temperature control. We performed benchmark runs typically for 10000 steps (20ps) with/without writing output any trajectory and coordinate files (Note that with no write trajectories and confout slightly increases the performance). For our tests, we used the “-pin on” and “-dlb yes” GROMACS flags, where “-pin on” stopped the kernel from moving processes between cores by locking the cores, and allowed dynamic load balancing to automatically run when the load imbalance was 5% or more, which is important for handling inhomogeneous systems. For optimal performance, we also tried mdrun −resethway and −maxh=0.05 options, which corrected the benching results. After these first test runs, the force fields for SP-A were taken into consideration for a total of 10 ns, or 5,000,000 time steps, in order to obtain reasonable interaction accuracy of SP-A within a water model.

### T-REMD device: JUWELS supercomputer

The JUWELS multi-petaflop supercomputer (54) is located at the Julich Supercomputing Centre (JSC, Germany). This is one of the most powerful computing resources available in Europe. It consists 2567 compute nodes (2511 CPU-only partitions and 56 Nvidia V100 GPU nodes), where the nodes are interconnected through Mellanox Infiniband high performance network architecture. The CPU-nodes are equipped with two Intel Xeon Platinum 8168 processors (base frequency of 2.7GHz), while GPU-nodes are fitted with the two 2.4GHz Intel Xeon Gold 6148 processors. Each GPU node contains four Nvidia V100 cards with 5120 CUDA cores. Note that the peak performance of the mentioned cluster is ~4,15 TF/s based on the Linpack Benchmark.

### T-REMD and classical graph cutting device: Bridges at the Pittsburgh Supercomputing center

The Bridges Supercomputer at the Pittsburgh Supercomputer Center has 752 Regular Shared Memory (RSM) nodes. Each of these nodes consist of 2 Intel Haskell CPUs with 14 cores per CPU, 9 AI-GPU nodes, each including 2 Intel Xeon Gold 6148 CPUs with 20 cores each and 8 NVIDIA Volta V100 GPUs. Because of GROMACS’ capability to improve performance through the use of GPUs, the AI-GPU nodes were used for the completion of OPLS, CHARMM, and AMBER force field implementations in T-REMD analyses on SP-A. These nodes were also utilized for the completion of the Goemans-Williamson interpretation of the MaxCut problem.

### Goemans-Williamson implementation

We applied the Goemans-Williamson algorithm by using the CVXGraph Algorithms Python package across the entire atom map of the protein. In this implementation of the algorithm, the atoms that were identified to be cut, were cut from the map, leaving the most energy-resilient atoms, and therefore the key binding sites on the protein.


(15) $$E[W]=\sum_{i<j}^{}\omega_{ij}\frac{\text{arccos}(\upsilon_{i}*\upsilon_{j})}{\pi }$$

Equation 4. Goemans-Williamson MaxCut algorithm (55): *E[W]* represents the expectation value of a node, *i* and *j* represent the two dimensions of node movement, *w* represent the weight of each node, and *v* represent the vector that the node produces itself.

We used the Bridges Supercomputing System to run this algorithm with PySpark used as the batching mechanism between nodes. Other than this addition, there were no additional changes made to the CVXGraph Goemans-Williamson algorithm used.

### Protein pruning by QAOA-based MaxCut to feed into modular binding/docking algorithms

**Figure 2:**
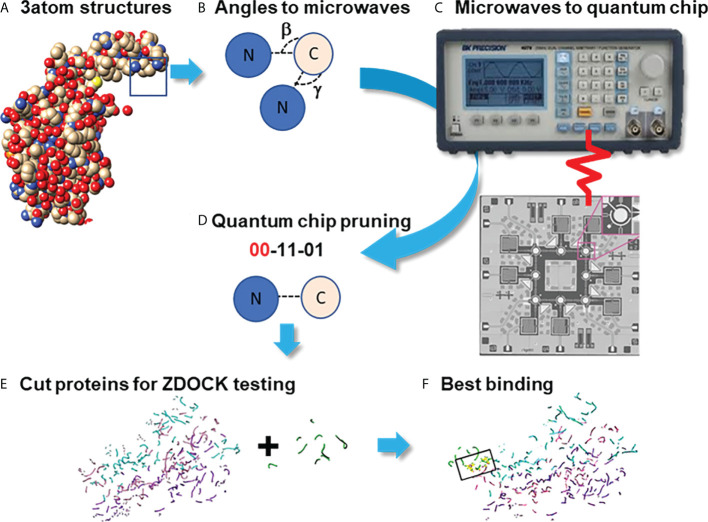
Pruning Program by utilizing the Quantum Approximate Optimization Algorithm (QAOA) on the Rigetti Quantum Processor. Process diagram for finding protein binding by Pruning. **(A)** Create graphs of 3 neighboring atoms each, with angles beta and gamma stored **(B)** Fourier-transform angles into frequencies to be placed on quantum chip by microwave. **(C)** Send microwaves to quantum chip. **(D)** Read results from quantum chip to determine which atoms to cut, with 00=Cut. **(E)** Summation of cut atom graphs to build reduced structures. **(F)** Binding studies between reduced structures with ZDOCK testing to identify the best binding sites.

Quantum computational graph cutting was necessary to overcome the poor computational scaling of the docking algorithms that make large scale protein structures prohibitively time- and compute-expensive. Additionally, ZDOCK's web server does not allow for large protein structure inputs. In this process, the weak potential bindings between atoms without polarized qualities (“topological minima or maxima” as described by Agarwal et al. (56) bound in low electronegative environments) were cut (**Figure 2**).

Using the QPUs as analogs for the atoms in the proteins, sets of three atoms each were mapped on qubits next to each other in placements topologically similar to the interaction space between the atoms themselves as identified by their PDB files. Then, either the Goemans-Williamson was implemented on the Bridges-AI cluster (https://www.psc.edu/resources/bridges-2/), or the QAOA-MaxCut package from Rigetti and Co. (https://grove-docs.readthedocs.io/en/latest/qaoa.html), were utilized to implement the MaxCut process on the 3-atom subgraph of those qubit positions on the Rigetti Aspen 8 QPU. The Rigetti Aspen 8 is a QPU device that operates using superconducting Josephson junctions to create a silicon based lattice structure of 31 qubits embedded onto a piece of gold and cooled to nearly 0°K through the use of helium based cooling chambers (https://patents.google.com/patent/US10050630B2/en).

**Figure 3:**
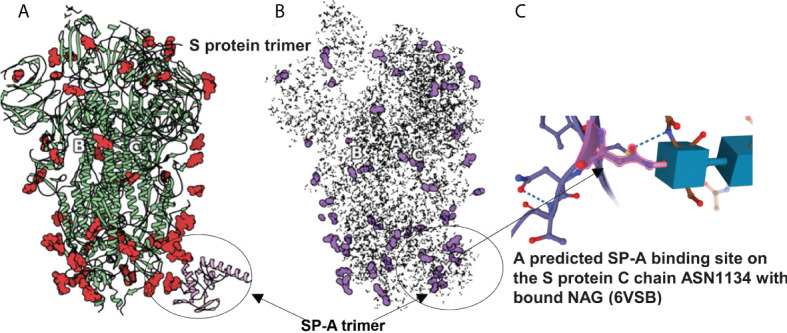
SARS-CoV-2 S protein (6VSB) modeling. **(A)**: The S protein trimer model (green ribbons representing chains A, B and C with red denoting glycan residues) and bound SP-A (pink). **(B)**: The final reduced S-protein-SP-A complex processed by our QAOA-based MaxCut protein pruning tool followed by ZDOCK docking (purple representing glycan residues and X marking the SP-A binding site. **(A, B)** were derived from the visualization software SAMSON. **(C)**: The amino acid ASN 1134 on the S protein C chain is identified as a likely candidate to mediate SP-A binding. ASN 1134 is outlined by dark pink and the blue cubes represent NAG glycosylation.

In the case of using Goemans-Williamson, the algorithm was implemented on the Bridges-AI cluster. However, the input and output processes for handling (e.g., with a Python file handler) with Goemans-Williamson were the same as for QAOA-MaxCut, and, at the end of these processes, basis states representing different qubits were cut from the graph at different probability levels. These basis states were translated to binary numbers according to the qubit and the flip state of that qubit, and were contextualized to identify the qubit that needed to be cut from the graph: 1s were accepted into the new graph, and 0s were eliminated. To find the best binding site in the best configuration, the highest probability basis states was assessed, and the atoms with positions that had 0 values within the basis states calculated were taken out from the overall list of protein atom positions. Lastly, these atom positions were then cross-referenced to the atoms they originally referred to in order to verify which atoms need to be part of a new PDB file representing only the best binding sites. Finally, this conversion took place using the Biopython software package. Once completed, the atoms that remained were rewritten into a Protein Data Bank (PDB) file (**Figure 3**) (47, 57).

### Measuring the effectiveness of SP-A models through docking using ZDOCK

In order to study the effectiveness of each model in determining tightest-bound binding sites, the models were made to bind with the SARS-CoV-2 spike protein in its open conformation (PDB: 6VSB). The binding between the reduced (pruned) structures was completed by the use of the software ZDOCK (University of Massachusetts Medical Center) (58, 59). Within this software, tightest-bound binding sites are determined through the closeness of a summation of Fourier transform of topological and desolvation energetic parameter scalars, and electrostatic values from CHARMM, for each atom in 6 dimensions. We checked the potential binding presence, affinity and locations for two complexes: the Spike protein and ACE2, as well as Spike protein and SP-A. Binding locations, ZDOCK affinity scores, and Root Mean Square Deviation (RMSD) scores between the top 2000 conformations were collected, assessed, and compared to each other and other literature data.

Because the binding capability was reflected as a scalar score value, a higher *Score_total_* value represents stronger binding. We compared the top 2000 conformations produced by ZDOCK for the AMBER, OPLS, CHARMM, and QAOA-MaxCut models and the tightest-bound binding candidates were reviewed against other SP-A results. RMSD values across the top 2000 conformations were then calculated to determine which model produces the highest accuracy conformations by the use of ZDOCK.

## Results

### SARS-CoV-2 S protein (6VSB) and SP-A (5FFR) trimer molecular pruning

The comparison of full protein structure and the reduced molecules is shown in **Figure 3**. The left panel (**Figure 3A**) depicts the known structure of Spike protein (as published in 6VSB). The reduced structure (**Figure 3B**) is a result of processing initial all-atom structures with MaxCut’s protein pruning. In this, the angles between every neighboring three atoms were Fourier-transformed into microwave frequencies and sent to a QPU that identified the chemically irrelevant atoms to eliminate based on their chemical topology (**Figure 2**). The structures produced by QAOA-MaxCut (**Figure 3** not right panel) were identical to those produced by Goemans-Willamson. This reduced all-atom structure has approximately one-third of the original atoms, leaving only those groups of atoms that represent the best binding sites for the protein; these are electrostatically more actively “charged” and more likely to be involved in the binding process. We confirmed that the remaining residues contained all the potential binding sites in the molecules as verified by the Universal Protein Resource (UniProt) database. QAOA-MaxCut was also compared to GROMACS using T-REMD using AMBER, OPLS, and CHARMM force fields (47, 57).

### SARS-CoV-2 S protein - SP-A complex formation

**Figure 4:**
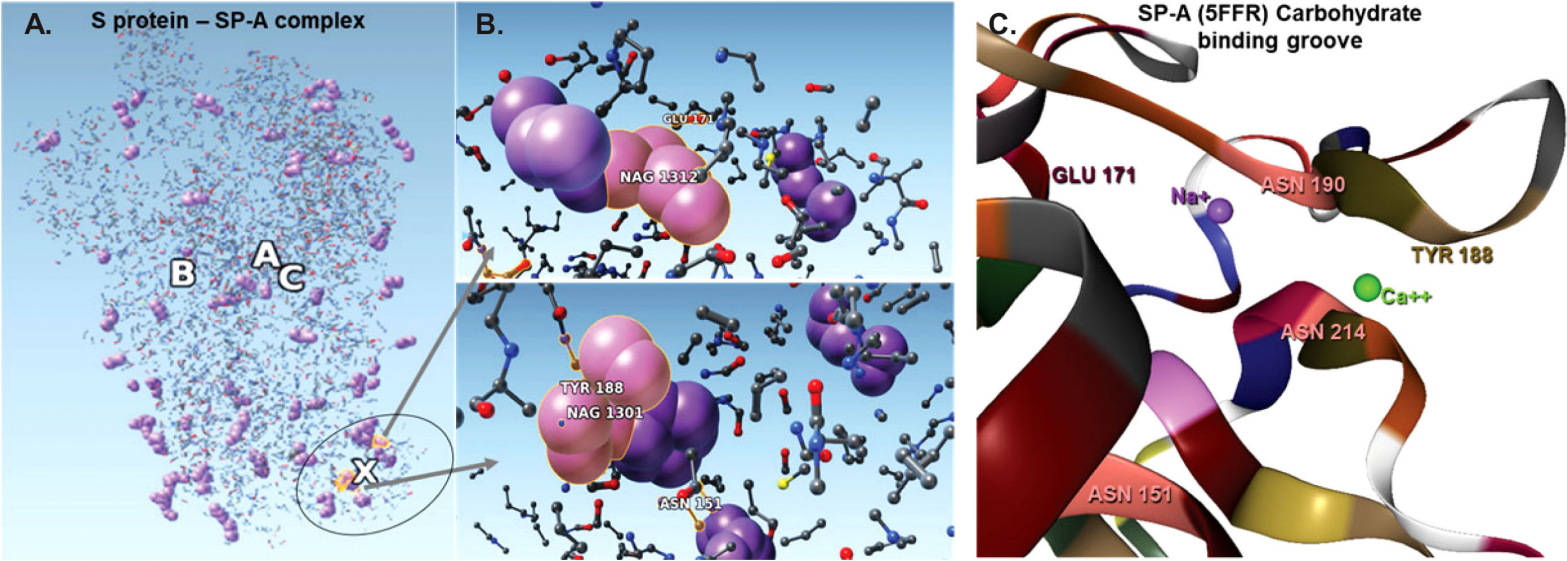
The complex resulting from docking of reduced structures of the S protein and SP-A with ZDOCK highlighting the top ranked binding sites on SP-A. **(A)**: Visualization by SAMSON after clashes/contacts with less than 2 Å distance were identified using the Chimera program. **(B)**: The top 5 binding sites are shown (ball and stick) highlighting bound NAG (purple space fill balls). SP-A and S protein amino acid residues are shown as a ball and stick. NAG1301 is bound to ASN1134 on the S protein and is shown in close proximity to ASN151 of SP-A. **(C)**: The SP-A carbohydrate binding grove showing the amino acids identified in the pruned complex including ASN 151 (pink) TYR 188 (khaki), GLU 171 (bordeaux) and Ca++ (green) and Na+ (purple). ASN 151 is clustered with ASN 214 and ASN 190 amplifying carbohydrate binding ability. The groove is flanked by TYR 188 and GLU 171 and harbors a Ca++ and a Na+ ligand. Presence of Ca++ is known to be required for carbohydrae binding. Atoms represented by the balls and stick are: (O): red; (C): grey; (Ca): green; (Na): purple; (S): yellow.

In order to prepare the SP-A protein for docking, we took the initial PDB file [model 5FFR (49)] from RCSB. Because 5FFR was designed for the assessment of SP-A lipid binding characteristics, it contained ions and phosphocholine to facilitate that binding. To avoid any unexpected influence from ions and phosphocholine, we removed them from the PDB. Then, we processed this SP-A’s PDB with the aforementioned protein pruning tool and received the reduced structure. Again, there was a significant change in atom numbers after using our QAOA-MaxCut based protein pruning tool: 5FFR (without ions and phosphocholine) has 1119 atoms and the reduced model has only 411 atoms. **Figure 4** shows the S protein and SP-A complex after docking of corresponding reduced PDB structures by ZDOCK. Surprisingly, while each of the GROMACS based models predicted SP-A binding to the open RBD of SARS-CoV-2 S protein, each of the graph cutting based methods predicted binding to S2 instead.

The bound residues identify the known carbohydrate groove of the SP-A including an ASN cluster (at positions 151, 190 and 214) flanked by TYR 188 and GLU 171. The structure also shows the close proximity of NA+ and Ca++. Ca++ is required for functional carbohydrate recognition by SP-A (**Figures 4B, C**).

### Characteristics of SARS-CoV-2 S protein-SP-A binding after QAOA-MaxCut pruning and root mean square deviation values

RMSD values indicate the average deviation between the corresponding atoms of two proteins evaluated for binding. Smaller RMSD values suggest greater similarity between the structures compared. The goal of designing improved algorithms has been to be able to find the best orientation between two structures that would result in the lowest possible RMSD. We calculated the RMSD values characterizing SARS-CoV-2 S protein-SP-A binding after QAOA-MaxCut pruning using ZDOCK scores.

**Table 1** shows the top 16 ranked conformations (out of 2000). The higher ZDOCK scores predict greater binding affinity between docking sites. Each specific docking site/conformation is identified by a 3-dimensional topological assignment denoted by x, y, and z coordinates.

**Table 1:** Top 16 ZDOCK scores, representing the electrostatic and geometric fit between protein residues, out of 2000 potential conformations between SP-A and SARS-CoV-2 Spike.

Conformation rank (of 2000)	X Docking Grid coordinate	Y Docking Grid coordinate	Z Docking Grid coordinate	ZDOCK Score
1	140	38	30	448.202
2	173	21	60	405.703
3	5	27	55	390.222
4	163	48	44	386.713
5	140	38	28	386.160
6	178	31	65	376.122
7	139	40	31	373.924
8	147	49	26	372.902
9	5	25	53	372.588
10	145	39	27	371.860
11	139	43	29	365.741
12	173	22	60	364.003
13	138	34	22	359.788
14	8	30	55	352.675
15	140	46	31	349.967
16	151	55	27	342.542

The central atom position between each conformations of the bound SP-A and the S protein is determined by the X,Y,Z coordinates that reflect the spatial localization differences.

**Table 2** shows the top 16 amino acids/ligands included in the binding between SARS-CoV-2 S protein and SP-A together with the atoms involved in the contact and the binding distance between these atoms. By studying the originally published 6VSB NAG glycosylated model of SARS-CoV-2 S protein, we found that many of the NAG residues located in the S2 fusion domain were predicted to be involved in SP-A binding. Notably, a group of NAGs (labeled in this model as 1301, 1302 and 1312) are identified as top candidates for binding. This is important because ASN molecules known to undergo post-translational glycosylation were also found in a cluster (at positions 1125, 1134, 1135) in the S2 fusion domain creating a “hot spot” for carbohydrate-lectin (add hyphen) binding. Indeed, from the SP-A side, the TYR at position 188 had the strongest potential binding with S protein NAGs, while ASN at position 151 also came up in multiple conformations. ASN 151, similarly to the SARS-CoV-2 S protein, forms a carbohydrate binding cluster with ASN 190 and 214 near to the Ca++ binding pore (**Figure 4**; **Table 2**).

**Table 2:** Top 16 binding sites between SP-A and SARS-CoV-2.

S Protein Chain; Amino Acid/Glycan residue; Contact Atom for Binding SP-A	Position of NAG or amino acid on the S protein (6VSB, published Feb 2020)	SP-A Amino Acid; Contact Atom for Binding the S Protein	Position of Amino acid on SP-A (5FFR)	Distance between both atoms (Å)
C; NAG; O	1301	TYR; C	188	1.308
C; NAG; O	1301	TYR; C	188	1.730
C; NAG; O	1301	TYR; C	188	1.905
C; NAG; C	1312	ASN; C	151	2.309
C; NAG; O	1312	ARG; C	197	2.079
C; NAG; O	1311	PRO; C	175	2.153
C; NAG; C	1312	GLU; C	171	2.455
C; NAG; C	1312	GLN; C	199	2.635
C; CYS; C	1126	SER; C	187	2.572
C; ILE; C	1130	SER; C	185	2.715
C; NAG; C	1302	TYR; C	188	2.620
C; NAG; C	1301	THR; C	189	2.653
C; NAG; C	1302	TYR; C	188	2.745
C; NAG; C	1302	TRP; C	213	2.749
C; NAG; C	1302	ASN; C	151	2.885
C; NAG; C	1311	PRO; C	175	2.809

The chain, the amino acid, and their respective atoms that are predicted to mediate binding between the S protein and SP-A according to the minimal distance that can be achieved between two atoms.

The average RMSD value calculated from 2000 ZDOCK binding conformations was 43.5 Å for the S protein - SP-A complex. This value is markedly lower than what we found for the average S protein - ACE2 RMSD, which was 272 Å following the same pruning and docking procedure. Using our QAOA-MaxCut protocol, the RMSD value was also lower than what we found by OPLS, AMBER, and CHARMM; these were 133.9 Å, 150.7 Å, and 173.1 Å, respectively, for assessment of SP-A- S protein binding. These results suggest that the S protein - SP-A complex forms with a high affinity, that it is likely a biochemically relevant configuration, and that the QAOA-MaxCut produced pruned structures are effective in predicting binding conformations.

## Discussion

Using a novel *in silico* approach we discovered potential binding sites between the SARS-CoV-2 S protein and SP-A, an immunoprotective lung collectin. We originally hypothesized that SP-A competes with ACE2 for the same binding sites. Our pruned molecular binding models indicated that SP-A is bound to the S protein with a similar affinity to that of ACE2 but in a different site in the S2 fusion segment. The amino acids involved in this interaction point to a highly glycosylated area of both molecules. Our hybrid quantum and classical computational study augments currently available structural and experimental results and highlights the importance of carbohydrate binding in the pathogenesis of SARS-CoV-2 infection.

The binding domain on the S2 segment we uncovered is not the most well-known of the S protein. Interference with this region, however, may affect viral fusion with the host cell membrane preventing viral entry and infection. Indeed, recent cryoEM, X-ray crystallography and membrane fusion assays show broad inhibition of the virus through areas other than the RBD (33–35, 60–62). Thus, binding by SP-A to the S2 region responsible for conformational destabilization of both the S1 and S2 segments may prevent viral entry to the host cell (9, 63). We found that SP-A would preferentially bind glycosylated sites on the S protein. Similarly to mannose binding lectin (MBL) and SP-D, carbohydrate binding by SP-A takes place in a specific pore in the carbohydrate recognition domain of the molecule in close proximity to a Ca+ ion. These collectin molecules have a high affinity to mannose (49). S protein is also a mannose-binding protein (20, 64). Interactions between SP-A, the S protein, and glycans may have functional significance in regulating protease access using glycan shields (8, 18).

Binding between the CRD of SP-A and carbohydrate residues on the S protein could also lead to opsonization and viral clearance by immune cells not bearing ACE2 receptors. SP-A binds to polysaccharides, phospholipids, and glycolipids on the surface of pathogens and also induces calcium-dependent aggregation of lipid vesicles (65). This binding is essential for the opsonization process resulting in the clearance and elimination of pathogens by phagocytes (22, 66, 67). SP-A-mediated phagocytosis is facilitated by collagen receptors such as calreticulin/CD91/LRP. In fact, SP-A can induce both anti- or proinflammatory immune cell responses by alternately ligating SIRP-α or calreticulin/CD91/LRP on the membrane of macrophages (68). Glycosylation alterations in SP-A can affect maturation, secretion, aggregation, and degradation of the molecule itself, although not usually through N-glycosylation sites (69). Thus, independent of ACE2 binding, SP-A recognition of carbohydrate moieties clustered on the surface of the S protein may drive pathogen clearance through opsonization (66) and protect from receptor-mediated internalization, increased inflammation, and systemic spread of infection (20).

Cell surface-bound lectin type receptors such as L-SIGN and DC-SIGN were also implicated in glycosylation-dependent S protein interactions with multiple cell types (70). Other cell surface receptors were also shown to facilitate alternative (ACE2-independent) viral entry to cells. Apart from the lectin receptors (that utilize carbohydrate moieties on the S protein), a group of integrin-type receptors also emerged as important players. Integrins can mediate viral internalization either through recognition of the arginin-glycine-aspartate (RGD) region of the RBD (arginin-glycine-aspartate) of the SARS-CoV-2 Spike protein (71–73), or independently of it (72). These alternative cell entry pathways are important in amplifying and spreading viral entry to structural cells. Thus, by binding to a highly glycosylated portion of the S2 region, SP-A may play an important protective role in ACE2-independent viral pathologies. It is notable that our investigation was based on utilizing a “fully glycosylated” model of the S protein (6VSB) to represent the SARS-CoV-2 S protein, as it had 44 of its 66 N-Acetylglucosamine (NAG) identified in experimental cryoEM microscopy work, not through computational placement (12). Since the initial release of the 6VSB model (that we used for identification of the NAG sites), the original NAG sites and identification numbers have been changed. It is important to bear in mind that most glycosylation sites on available models are arbitrarily added to proteins and may not always be an accurate reflection of reality. Our observations therefore warrant additional *in silico*, *in vitro* and *in vivo* investigations to verify and identify further mechanistic details and the specific clinical and pathological significance of carbohydrate-based SP-A – SARS-CoV-2 interactions.

Along the same line, the most naturally occurring configuration of SP-A is an octadecamer (22, 66) but our *in silico* predictions were performed using an available trimeric neck-CRD (5FFR) model of the molecule. SP-A (encoded by two genes SP-A1 and SP-A2 into largely identical 35-kD peptides) has a similar structure to MBL, SP-D, and C1q. Six of the SP-A homotrimers form an octadecamer “bouquet” of unidirectionally positioned molecules composed of a carboxy-terminal C-type lectin domain, a coiled-coil neck region, a collagen tail and an amino-terminal domain (19, 22, 26, 28). However, when complexed with SP-B, lipids or detergents (65) or under inflammatory conditions, SP-A loses its geometric and topological octadecamer features and structurally reforms into smaller oligomers (i.e., it falls apart) (74). Given that to date no experimentally determined SP-A structural model that includes all of its amino acids exists, *in silico* results using current truncated models should be carefully interpreted. Further studies will be necessary to determine how higher order oligomerization of SP-A is regulated and how it affects binding characteristics especially interactions between SP-A and the S protein.

Our computational approach used a shared logical model from the quantum chemistry of these proteins, including the modeling of their binding/docking after computationally pruning targeted sites to reduce the size of the proteins and the number of conformations to be analyzed. We used a combination of an electronegativity mapping software that performs QAOA-MaxCut functions, followed by ZDOCK assessment of binding between the SARS-CoV-2 S protein and SP-A. This novel approach allowed to complete top binding site determination in a more rapid manner than the widely used GROMACS program, while providing similar binding sites that OPLS, QAOA-MaxCut, and AMBER did. Quantum algorithms can utilize superpositions of each state and entanglement between states to produce strong cut probabilities nearly instantaneously for multiple atoms, while classical processing devices must complete these tasks serially for each atom. This allows for a slimmer algorithmic approximation that does not require multi-axial force calculations or higher order functions. The steps completed by GROMACS to properly characterize the forces for each atom are additive amongst the atoms and then additive amongst each pair of atoms, creating a computational complexity of *O*(*n* ^2^) for each atom (75). Goemans-Williamson, while it requires lower dimensionality, has a complexity of *O*(*n* ^2^ *logn*) due to its arccos term (76). Thus, QAOA is naturally the fastest algorithmic implementation, with an expected performance in the *O*(*logn*) regime.

Surprisingly, while each of the GROMACS based models predicted SP-A binding to the open RBD of SARS-CoV-2 spike, each of the graph cutting based methods predicted binding to S2 instead. This discrepancy prompted us to compare the programs we used. Regarding the accuracy of the configurational spaces, GROMACS was the only software that effectively captured the effects of simulation with a saline solution on the protein in the form of direct coordinate shifts, and initialization of a water model to ensure neutral solvency. However, this process, which leans heavily on atom-by-atom calculation of Coulombic forces, scaled up exponentially with squared time steps for each added atoms when it came to assessing protein-to protein interactions. To be able to feasibly perform our study, a cap at 5,000,0000 time steps, or 50 ns, had to be implemented on both the BRIDGES and JUWELS clusters limiting the ability to precisely simulate protein movement dynamics (57). Further, we compared the top docking score, docking pose (conformation and orientation), and Root Mean Square Deviation (RMSD) across OPLS, AMBER, CHARMM, and the QAOA-MaxCut-prepped SP-A models. We found that the docking values created with the new graph cut model had lower deviation across all final conformations than those found by GROMACS, and the top binding sites identified on SP-A were on the same residues by all the programs we studied. These results suggested that the S2 - SP-A complex forms with a high affinity, that it is likely a biochemically relevant configuration, and that the QAOA-MaxCut produced pruned structures are effective in predicting binding conformations.

Nonetheless, it is important to address the question whether the discrepancy in binding site prediction between the GROMACS and QAOA-MaxCut programs could be due to the elimination of some important amino acids by the latter. QAOA-MaxCut is a state-of-the-art method to approximating a solution to MaxCut, a problem-space characterized by the maximum size cut within a graph of nodes and edges that cannot be completed in polynomial time on a classical computer. Critically, QAOA-MaxCut studies atomic graphs of only three atoms in every instance of cutting one. The program finds maximum cut values based on the angles within the graph and evaluates the ability of the algorithm on quantum hardware to reach the lowest possible cost values, or combined bias values, for each potential cut. The graphs we fed the algorithm in this study had the bond angles of each atom within the 3-atom sets. As electronegativity and bond angles are directly proportional, the most electronegative atom with the largest bond angle within a set was cut. Important amino acids would not be eliminated in this atomic level modeling. Additionally, we confirmed that the remaining residues contained all the potential binding sites in the molecules as verified by the Universal Protein Resource (UniProt) database for both SP-A and the SARS-CoV-2 S protein. On SP-A, UniProt identified a glycosylation site at amino acid 207, which in our model would be amino acid 214 (5FFR), included in the ASN cluster we identified. As we discussed above, in our interpretations we need to carefully take into account the discrepancies in the amino acid and glycan ligand numbering due to structural file differences between available published structures.

Our computational model while, providing improved accuracy (low RMSD) and efficiency (reduced computational time) in assessing protein-protein binding sites between SP-A and the SARS-CoV-2 S protein similarly to other currently available algorithms, could not provide insights to the molecular dynamics of the bindings. Molecular dynamics simulation packages such as GROMACS are based on the application of classical mechanics models to study physical systems at the atomic level. Regardless of the software, they include force computation with van der Waals, electrostatic (Coulomb), and various bonded and non-bonded terms to provide a projection of laboratory experiments with potentially greater detail albeit still as an approximation [reviewed in detail by Khan et al.(77)]. Importantly, protein folding, the process necessary to assume biologically meaningful ligand-receptor interactions, is estimated to take at least a microsecond (77), making accurate modeling of these currently beyond the reach of available computational approches. How molecular dynamics simulation can be accelerated, however, is an exciting area of investigations in the computational field.

## Data availability statement

The raw data supporting the conclusions of this article will be made available by the authors, without undue reservation.

## Author contributions

SA performed the study and wrote the initial draft. KM and SS expanded the draft to include algorithmic details and binding sites on glycosylation and serine proteases. AH and SS conceived the idea for the study and directed the work. AH and KM edited the manuscript. All authors contributed to the article and approved the submitted version.

## Funding

SS: Pittsburgh Supercomputer Center startup award from the Extreme Science and Engineering Discovery Environment (XSEDE). AH: Chester Robbins Endowment for pulmonary research, UC Davis; 27IR-0053C Tobacco Related Disease Research Program, UCOP

## Acknowledgments

We would like to thank Vaibhav Gupta and the development team at Iff Technologies for building and improving upon the software; Rigetti and Co for providing credits to use their platform and their employees, Mark Skillbeck and Tom Lubowe, for providing software and hardware troubleshooting help. Angela Linderholm, Melissa Teuber, Pedro Hernandez, and Nikita Mohapatra of the Haczku Lab for weekly discussions on presentation and interpretation of the results, and Dr. Armen Poghosyan, Bioinformatics Group Leader at the International Scientific and Educational Center, the National Academy of Sciences of the Republic of Armenia (NAS RA) for fruitful discussions around accurate modeling of SP-A and modeling support.

## Conflict of interest

SA was working for Iff Technologies developer of the graph pruning algorithm. KM and SS are current shareholders and employees of Iff Technologies.The remaining author declares that the research was conducted in the absence of any commercial or financial relationships that could be construed as a potential conflict of interest.

## Publisher’s note

All claims expressed in this article are solely those of the authors and do not necessarily represent those of their affiliated organizations, or those of the publisher, the editors and the reviewers. Any product that may be evaluated in this article, or claim that may be made by its manufacturer, is not guaranteed or endorsed by the publisher.

## Abbreviations

ACE2, Angiotensin Converting Enzyme 2; ARG, Arginine; ASN, Asparagine; CHARMM, Chemistry at Harvard Macromolecular Mechanics; COVID-19 Coronavirus Disease Pandemic 2019; CRD, Carbohydrate Recognition Domain; Fc, Crystallizable Fragment; GLN, Glutamine; GLU, Glutamic acid; HAT, Human Airway Trypsin-like protease; HIS, Histidine; LEU, Leucine; MASP, Mannose Binding Lectin Associated Serine Protease; MBL, Mannose Binding Lectin; NET, Neutrophil Extracellular Traps; NHS, National Health Service, United Kingdom; NISQ, Near Intermediate Scale Quantum; PAR4, Protease-activated Receptor 4; PHE, Phenylalanine; PRO, Proline; QAOA, Quantum Approximate Optimization Algorithm; RMSD, Root Mean Square Deviation; RSV, Respiratory Syncytial Virus; S1, Subunit of the SARS-CoV-2 Spike that is found at its tip; S2, Subunit of the SARS-CoV-2 Spike that is bound to the rest of the virion; SARS CoV-2, Severe Acute Respiratory Syndrome Coronavirus 2; SER, Serine; SIRPα, Signal Regulatory Protein-alpha; SP-A, Surfactant Protein-A; SP-B, Surfactant Protein-B; SP-C, Surfactant Protein-C; SP-D, Surfactant Protein-D; S Protein, SARS-CoV-2 Spike Protein; THR, Threonine; TMPRSS2, Transmembrane Protease Serine 2; TRP, Tryptophan; TTSP, Type II Transmembrane Serine Proteases; TYR, Tyrosine; UNK, Unknown/Unlabeled; VAL, Valine; ZDOCK, Docking Program based on the Fast Fourier Transform algorithm developed by the Zheng Lab, at the University of Massachusetts, Amherst.

## Glossary

**Table:**

ACE2	Angiotensin Converting Enzyme 2
ARG	Arginine
ASN	Asparagine
CHARMM	Chemistry at Harvard Macromolecular Mechanics
COVID-19	Coronavirus Disease Pandemic 2019
CRD	Carbohydrate Recognition Domain
Fc	Crystallizable Fragment
GLN	Glutamine
GLU	Glutamic acid
HAT	Human Airway Trypsin-like protease
HIS	Histidine
LEU	Leucine
MASP	Mannose Binding Lectin Associated Serine Protease
MBL	Mannose Binding Lectin
NET	Neutrophil Extracellular Traps
Extracellular Traps	
NHS	National Health Service United Kingdom
NISQ	Near Intermediate Scale Quantum
PAR4	Protease-activated Receptor 4;
PHE	Phenylalanine
PRO	Proline
QAOA	Quantum Approximate Optimization Algorithm
RMSD	Root Mean Square Deviation
RSV	Respiratory Syncytial Virus
S1	Subunit 1 of the SARS-CoV-2 Spike that is found at its tip
S2	Subunit 2 of the SARS-CoV-2 Spike that is bound to the rest of the virion
SARS CoV-2	Severe Acute Respiratory Syndrome Coronavirus 2
SER	Serine
SIRPa	Signal Regulatory Protein-alpha
SP-A	Surfactant Protein-A
SP-B	Surfactant Protein-B
SP-C	Surfactant Protein-C
SP-D	Surfactant Protein-D
S Protein	SARS-CoV-2 Spike Protein
THR	Threonine
TMPRSS2	Transmembrane Protease Serine 2
TRP	Tryptophan
TTSP	Type II Transmembrane Serine Proteases
TYR	Tyrosine
UNK	Unknown/Unlabeled
VAL	Valine
ZDOCK	Docking Program based on the Fast Fourier Transform algorithm developed by the Zheng Lab, at the University of Massachusetts, Amherst
